# Development and Updating of Clinical Practice Guidelines—The Necessity of International Collaboration

**DOI:** 10.1002/gin2.70077

**Published:** 2026-06-14

**Authors:** Johanna H. van der Lee, Irina M. Mostovaya, Johannes J. Duvekot, Robbert J. Ensink, Klaartje Spijkers, Margreet Pols, Teus A. van Barneveld

**Affiliations:** ^1^ Knowledge Institute of the Dutch Association of Medical Specialists Utrecht the Netherlands; ^2^ Department of Obstetrics and Gynecology Erasmus University Medical Center Rotterdam the Netherlands; ^3^ Department of Ear Nose and Throat Surgery, Gelre ziekenhuizen, Apeldoorn Zutphen the Netherlands; ^4^ The Netherlands Patient Federation Utrecht the Netherlands; ^5^ Dutch Association for Clinical Physics Utrecht the Netherlands

## Abstract

Development and updating of clinical practice guidelines requires substantial resources. The Knowledge Institute of the Dutch Association of Medical Specialists has extensive experience in supporting the development of medical specialist guidelines in close partnership with the Netherlands Patient Federation, and is a long standing and active member of the Guidelines International Network. Based on the evaluation of an international pilot project in obstetrics, we propose a model for international collaboration: jointly identifying and prioritizing shared clinical questions, dividing the development of evidence summaries across countries, and exchanging these summaries so national guideline groups can develop context‐specific considerations and recommendations. International collaboration can improve guideline quality, make optimal use of available scientific knowledge, and increase efficiency. With this paper, we call on guideline development organizations in other countries to collaborate in exchanging evidence summaries, so that all parties can benefit from more efficient guideline development.

## Summary

To improve the efficiency of clinical guideline development, we propose an international collaboration model in which evidence summaries are shared. Positive experiences and challenges encountered in a pilot project are described, with recommendations for international standardization of procedures such as mandating multidisciplinary panel members, involving patient representatives and the joint prioritization and formulation of clinical questions.

## 1 What Is the Issue?

Keeping national guidelines up to date demands more time, funding, and expertise than many countries can supply. In the Netherlands, updates to specialist guidelines are financed through the Dutch Medical Specialists Quality Fund (abbreviation in Dutch SKMS). This funding allows about 10% of the guidelines for medical specialist care to be updated per year. As a result, only the most urgent clinical questions can be addressed.

Meanwhile, the growing complexity of guideline development—driven by evolving GRADE methodology and the rapidly increasing volume of scientific literature—intensifies the workload.

These challenges are not unique to the Netherlands. Organizations worldwide face similar constraints, as highlighted by initiatives such as the Alliance for Living Evidence (ALIVE).

One option would be to rely on guidelines developed by international medical societies. However, these guidelines often lack multidisciplinary input and rarely incorporate patient perspectives, and they usually require substantial adaptation at the national level.

In contrast, a shared evidence synthesis model reduces duplication of effort while allowing for national differences. This paper presents the experiences and lessons from an international pilot project in obstetrics and offers recommendations for future cross‐country collaboration.

## 2 Who We Are

The Knowledge Institute of the Dutch Association of Medical Specialists supports the development and updating of national clinical practice guidelines in the Netherlands. The public Dutch guidelines database contains more than 10,000 clinical questions (in about 650 guidelines). Evidence summaries in recent updates are written in English, and most Dutch content can be automatically translated through most web browsers.

For the development of guidelines we use the GRADE methodology, and our guidelines comply with the AGREE II criteria. Important aspects are a comprehensive representation of interest‐holders and addressing potential conflicts of interest. We use the Dutch Code for the prevention of improper influence due to conflicts of interest. We aim for inclusion of all relevant interest‐holders, in particular patient representatives, in the guideline panels and in the process of defining clinical questions that need to be addressed or updated.

Our partner the Netherlands Patient Federation is an umbrella organization representing over 200 Dutch patient organizations active at the national level, most of them also on patient participation in guideline development. Over the past decade, we have built a strong collaborative model to include the patient perspective: what experiences do they have and what are their needs for better outcomes of care.

The need for updates continues to outpace available funding and capacity. This tension is a key reason why international collaboration is increasingly necessary.

## 3 Pilot Project in Obstetrics

To explore this collaborative model, a pilot project was launched in 2018 by the Dutch Society of Obstetrics and Gynaecology (NVOG) together with the Knowledge Institute and with professionals and patient representatives from Belgium, Germany, and the United Kingdom.

Panel members were mandated by national gynaecological societies. A jointly approved collaboration agreement and “roadmap” guided the process.

Eight clinical questions were selected, covering topics such as Prevention and management of umbilical cord prolapse, Corticosteroids and planned caesarean birth (34 ^+0^ to 36^+6^ weeks of gestation, 37 ^+0^ to 39^+6^ weeks of gestation, second course of corticosteroids), Antenatal care after IVF or ICSI, and Induction of labour after IVF or ICSI.

Literature searches were conducted by an information specialist from the Knowledge Institute. Study selection was performed independently by two panel members using Rayyan. Evidence summaries and GRADE conclusions were written by a methodologist of the Knowledge Institute. Pairs of panel members drafted considerations using the Evidence to Decision framework [1]. If necessary, the considerations contained a paragraph on the current situation in each country. The full panel reached consensus on recommendations.

During public commentary, two recommendations related to antenatal corticosteroid use (34–37 weeks) were revised for one country by the national society due to differing national practices.

Below, we describe the lessons learned from this pilot project, also documented in an evaluation report, divided into positive experiences and challenges.

## 4 Positive Experiences

Although several challenges were encountered, all participating parties expressed positive views on the opportunity to collaborate within a structured guideline development process and on the methodological support provided by the Knowledge Institute. Panel members particularly valued the international exchange of perspectives.

Early in‐person meetings played an important role in building mutual trust and fostering constructive collaboration; informal social interactions, such as shared dinners and drinks, were also important for team building. When the COVID‐19 pandemic later required a transition to online meetings, collaboration remained effective, partly because these personal connections had already been established. The majority of the panel members joined a follow‐up project, demonstrating sustained enthusiasm [2].

## 5 Challenges

The project also faced several challenges which can be addressed in future projects.

Securing multidisciplinary participation was difficult; despite considerable efforts, only one midwife participated. Invitations to paediatric organizations to mandate a neonatologist remained unanswered, leading to challenges during the commentary phase.

Procedures for involving patient representatives varied across countries. Only the Netherlands had structured patient participation from the start. For future projects it is recommended that patient representatives from all countries must be engaged from the outset, with adequate funding and clear guidance.

In this pilot, clinical questions were chosen by the chair and one member, without a broad consultation. Future initiatives should use a standardized, participatory agenda‐setting process in which patient involvement is secured.

Countries lacked consistent systems for collecting interest‐holder comments. A standardized, cross‐country review procedure is needed to ensure transparency and completeness. Although the panel members in the pilot reached consensus on the recommendations, the national society in one of the countries disagreed, and adapted the recommendations to make them applicable to the national context. We think that individual countries should always be free to formulate their own recommendations. In no way does this diminish the advantages of international collaboration.

## 6 Moving Forward

This pilot showed that international collaboration in guideline development is greatly appreciated by panel members, sharing evidence summaries is feasible and highly valuable, but there are several challenges that need to be overcome. To move forward, we need to develop and coordinate international procedures for mandating multidisciplinary panel members, involving patient representatives from the start, and conducting the (national) review and endorsement phases. Also, we need to standardize how clinical questions are jointly formulated, including PICOs and outcome selection (with Minimal Important Differences), in order to use the GRADE methodology consistently. In our opinion, countries should be free to either adopt jointly formulated recommendations, or to write their own considerations and recommendations shaped by national contexts, acknowledging differences in health‐care organization, reimbursement policies and availability of medical equipment and expertise.

## 7 Invitation to Collaborate

In a future project, we plan to apply the lessons learned and develop standardized procedures for international collaboration in guideline development. Starting with three specialties—Obstetrics/Gynaecology, ENT surgery, and Orthopaedics—we will build European collaborations, develop or update guidelines jointly with the possibility to write considerations and recommendations at the national level. In this project we will further explore ways to standardize organizational procedures for cross‐country projects and create a clear framework for meaningful patient involvement.

We believe that by working with guideline developers worldwide—who understand their national contexts best—we can make international collaboration the norm rather than the exception.

We warmly invite organizations interested in this collaborative approach to join us.

## Author Contributions

**Johanna H. van der Lee:** conceptualization, writing – original draft, writing – review and editing. **Irina M. Mostovaya:** conceptualization, writing – review and editing. **Robbert J. Ensink:** writing – review and editing. **Margreet Pols:** writing – review and editing. **Teus A. van Barneveld:** conceptualization, writing – review and editing.

## Funding

The authors have nothing to report.

## Conflicts of Interest

The authors declare no conflicts of interest.

## Data Availability

The data that support the findings of this study are openly available in richtlijnendatabase at https://www.richtlijnendatabase.nl.
